# INtelligent toolkit for reconnaissance, assessments and prehospital support in Perilous InciDents: a realistic experiment in prehospital environment

**DOI:** 10.1186/s12913-024-11786-3

**Published:** 2024-11-01

**Authors:** Ana María Cintora-Sanz, Patricia Blanco-Hermo, Soledad Gómez-De la Oliva, Rozenn Marechal, Olivier Balet, Patricia Gonzalez-Rico

**Affiliations:** 1 Prehospital Emergencies Medical Service. Madrid Region (SUMMA112) Antracita, 2 Bis, Madrid, 28047 Spain; 2 CS GROUP France, 6 rue Brindejonc des Moulinais, Toulouse, Cedex 5, 31506 France

**Keywords:** Pre-hospital Care, Decision support techniques, Unmanned aerial devices, Ecological parameter monitoring, Robot enhanced procedures

## Abstract

**Background:**

First responders, when arriving at a disaster, need a rapid analysis of the environment in which they are going to operate, as they have to assess the conditions surrounding potential victims and neutralize any risks that may exist.The EU-funded INTREPID develops a new technology platform to assist first responders when arriving on the scene of a disaster. The project INTREPID aims to support safer operations in the form of more efficient, fast, and safe disaster site assessments. The objective of the study is to implement new technologies into rescue operations to facilitate and improve situational awareness and operation management capabilities to save lives.

The focus of the study is relevant to the field of mass casualty incident management and disaster, as proper communication is extremely relevant in the management of catastrophes.

**Method:**

The first phase of the project started with a qualitative methodology SCRUM, for catching the end user’s feedback and requirements to design the interface platform. It was developed a platform to support first responders in disasters areas improving the 3D scanning and analysis of disaster areas. This platform is based on the concepts of intelligence amplification and eXtended Reality, with hololens, drones and robots. The project continued with a β phase in which the platform with all tools integrated were tested in simulated mass casualty disasters.

**Results:**

These technologies are tested in different disaster scenarios: A flooded subway stop in Stockholm, an accident in the chemical industry in Marseille, and a man-made explosion in a hospital in Madrid. Through this platform, first responders can immediately initiate operations without exposing personnel to potential harmful risks without specialized equipment, with all important information shared and coordinated, among all responders, whether they are security, firefighters, or emergency health professionals.

**Conclusions:**

The performance pilots and the questionnaire results validated the effectiveness and usability of the final version of the INTREPID platform and tools.

**Supplementary Information:**

The online version contains supplementary material available at 10.1186/s12913-024-11786-3.

## Introduction

Ground-breaking technological progress in the 21st century has triggered the increased use of new technologies in recent years in catastrophic incident situations, with a particular impact on the healthcare field [1, 2].

First responders must deal with pressing and dramatic challenges in a chaotic, dynamic and dangerous environment while locating and rescuing victims and neutralizing threats as soon as possible. They must make urgent decisions contemplating the possibility that the area may be large, complex, hostile, and with many unsafe areas to explore. Lack of reliable information and profound uncertainty present serious obstacles to a rapid and effective response. Rescue operations must be initiated to save as many survivors as possible [3, 4].

**Fig. 1:**
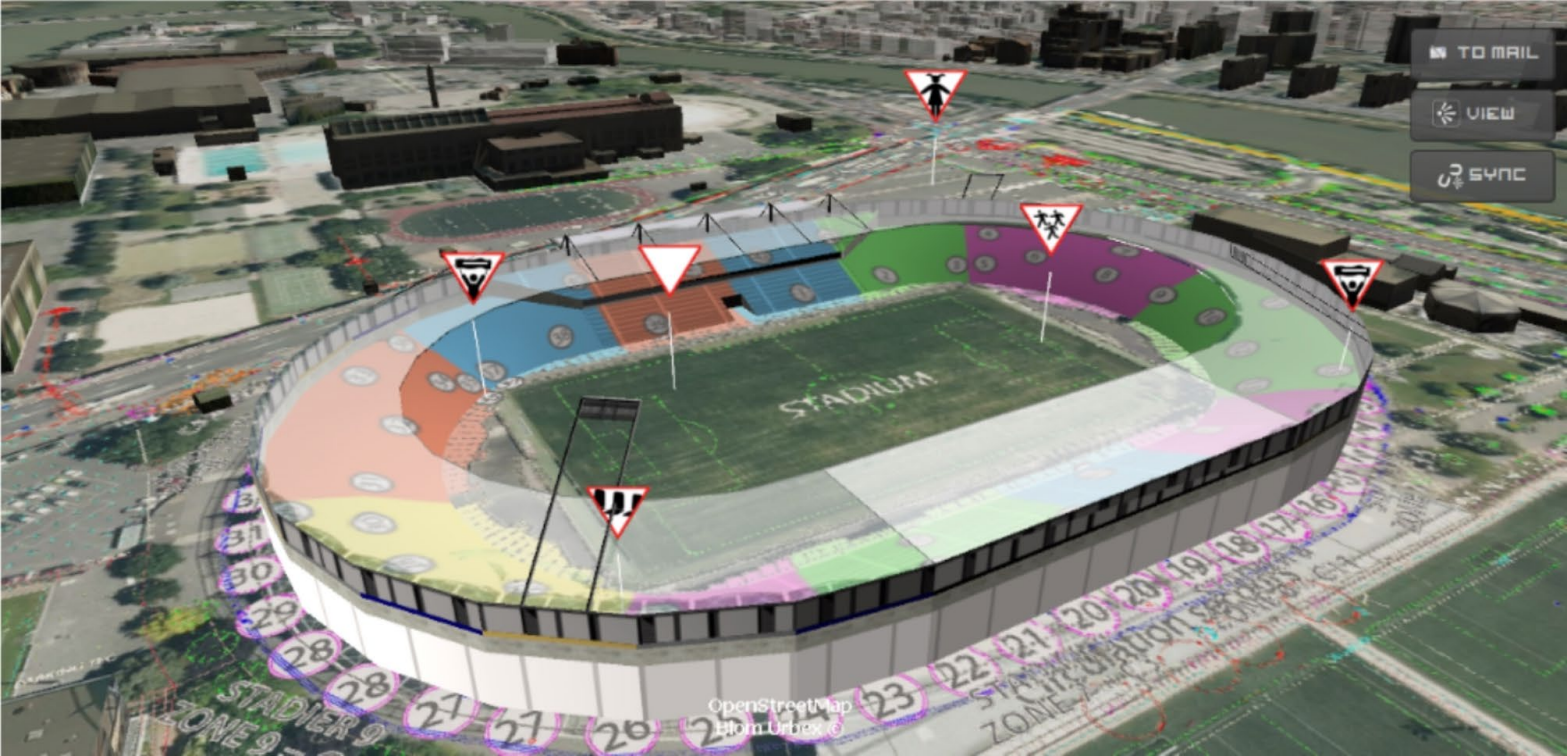
Information captured from the field placed in the INTREPID Platform’s improving situational awareness

In this regard, it must be borne in mind that, to enable these operations to be effective and safe, robots are being geared to assist emergency services by providing unprecedented situational awareness of disaster areas. Unmanned Aerial Vehicles (UAV) and remotely operated Unmanned Ground vehicles (UGV) have been used to generate three-dimensional (3D) maps of disaster areas [5, 6], search for victims [7, 8], and assess risks [8, 9], (see Fig. 1), the INtelligent Toolkit for Reconnaissance and assessments in Perilous InciDents (INTREPID) H2020 Research Project [10], aim this purpose and develops these cybertools.

### Background and project context

At the beginning of disasters, the most important difficulties for first responders are communication, coordination and security. Mainstream media are often affected by the disaster and it is necessary for those involved to coordinate effectively on the ground, with concrete and accurate information about what is happening, to resolve the incident and minimise the damage [11, 12]. End users involved in a disaster require rapid analysis of the environment in which they are working, as they have to deal with adverse conditions in their efforts to rescue victims and neutralize risks. There is a need to optimise the management of disasters, whether man-made, chemical, biological, radiological or nuclear. There is no homogeneous communication system that shares disaster information across all the emergency systems that intervene to solve the disaster situation, whether man-made or from natural hazards [13–15] .

Until now, drones and robots have been limited to providing information in scenarios where first responders could not enter [16]. They have been used in disasters mainly for logistical support and visibility of scenarios [5], but they have much more versatile features. The application of drones in emergencies was structured by Mohd [12] in 4 main competences: (1) mapping of disaster areas, which represents the main contribution, (2) search and location of victims (i.e. maritime rescue, ) (3) transport of rescue elements (automatic defibrillator, etc.) and (4) training. With INTREPID we optimise the performance of these cyber assistants, fostering synergy of robots with drones providing information and support to the emergency services, helping in the triage and assistance of victims with indoor screening Chemical-Biological- Radiological-Nuclear (CBRN) risk areas.

Furthermore, the INTREPID project targets to assist first responders by providing a unique platform to improve 3D mapping and disaster site assessment. This platform is based on Augmented Intelligence (AI) and Extended Reality (XR) concepts, coworking with intelligent cybernetic assistants ( “UAV” and “UGV”), as well as innovative positioning technologies and indoor networking capabilities.

Augmented intelligence is a subsection of Artificial Intelligent machine learning developed to enhance human intelligence rather than operate independently of or outright replace it. It’s designed to do so by improving human decision-making and, by extension, actions taken in response to improved decisions [17]. XR [18] is an umbrella term for any technology that alters reality by adding digital elements to the physical or real-world environment to any extent and includes augmented reality (AR), mixed reality (MR), and virtual reality (VR) [19].

Through this platform, first responders can get to work immediately without having to wait for specialized teams or for the area to be fully secured. All the information collected is aggregated and merged by the platform.

**Fig. 2:**
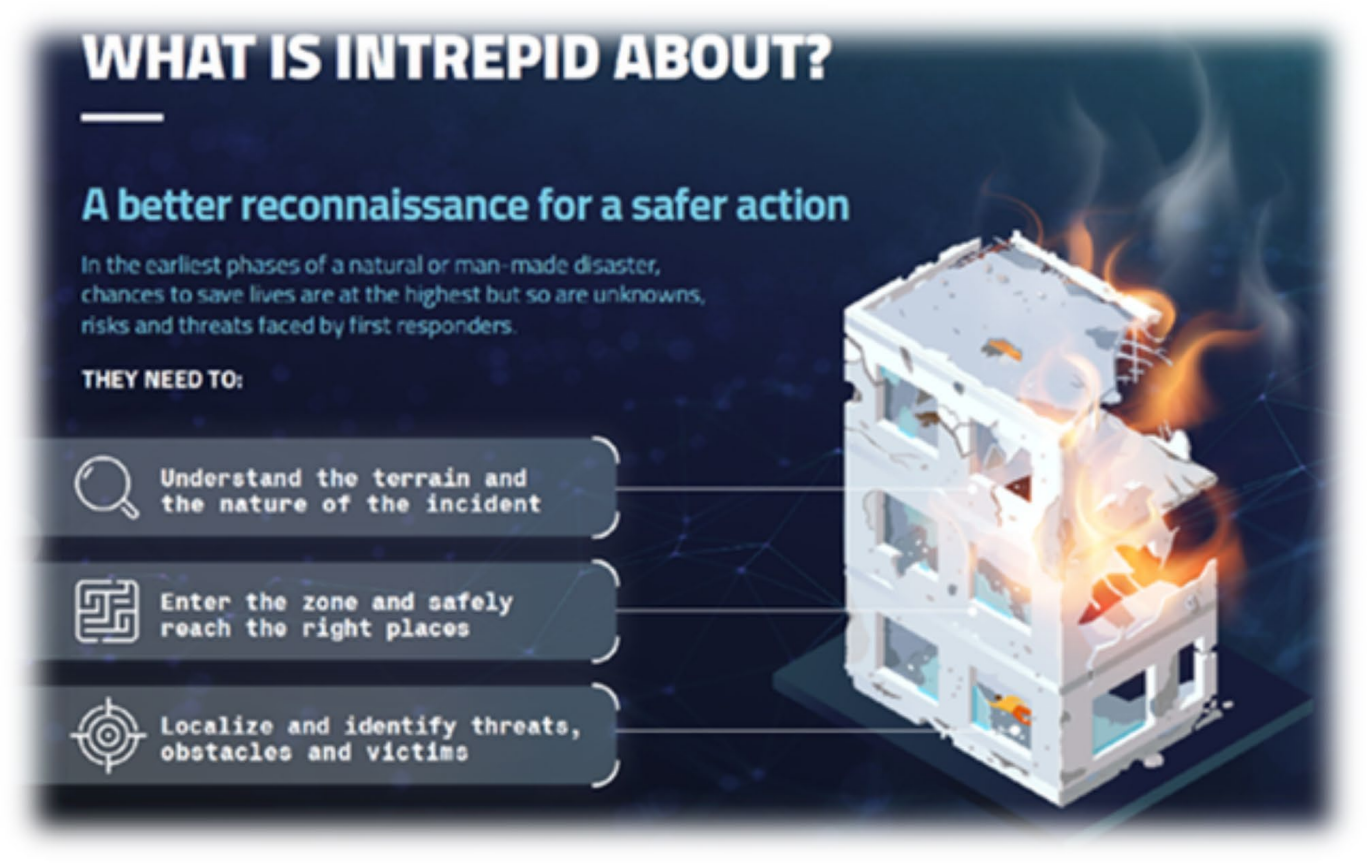
The infographic of INTREPID’s project focus

The resulting Digital Twin of the disaster site and ongoing situation provides indispensable and highly legible situation awareness enabling fast and targeted multidisciplinary response in the form of earlier, more effective, and safer operations, not only for first responders but also for victims (see Fig. 2).

INTREPID project [20] is co-funded by the European Commission in its Security call. It has had a duration of 3 years, from October 2020. It has been formed by a consortium of leading research centers, coordinated by CS Group, an industry with a leading position in the security market, SMEs, as well as a diverse representation of emergency institutions: health services, law enforcement agencies from different countries and fire brigades. The consortium is made up of 7 European Countries, 7 End-users or First Responders, 7 Major Research and Technology Developers, and 3 Experts in Crisis, Legal, Ethics, and Communication.

INTREPID follows a user-centered methodology involving many first responders, an International Advisory Board, and an Open User Group that will ensure diversity. Social, ethical, and legal constraints are carefully considered during the life of the project.

## Objectives

INTREPID’s overall objective is to develop a modular, secure and scalable technology platform based on the Crimson crisis management deck [21] to facilitate the complete and safe exploration and assessment of hazardous, potentially inhabited sites after a disaster.

These general objectives are achieved through the development of the following specific objectives.

1. Rapid and safe assessment of the scene by determining the location of obstacles and other risk points.
  - Design tools that can determine the safest entry and exit points to the intervention area for first responders.
  - Improve tools to design a safe route for first responders to access victims within the intervention zone.
  - Develop cyber-assistants that explore digital pre-disaster scenario models, enriching them with data captured in the disaster area, and being able to generate optimal and safe trajectories for the movement of first responders or cyber-assistants.
  - Test tools to optimize the exploration of a scenario as well as the assessment of a situation involving first responders.
2. Create tools that facilitate the assessment of potentially hazardous habitable spaces.
  - Improve tools that facilitate the detection of chemical substances.
  - Test cyber assistants to design a real-time automatic 3D mapping of the environment to update the digital twin of the site.
3. Determinate in real-time the position of first responders and vehicles inside the intervention zone (hot zone) both in low visibility conditions and in areas inside or outside buildings.
  - Use an Interface that improves the accuracy of the Global Positioning System (GPS) positioning of first responders indoor/outdoor.
  - Develop a communication platform that facilitates the real position of the cyber-assistants intervening in the disaster area.
  - Specify in real-time the position and clinical situation of victims for all stakeholders of the disaster area.
4. Design of a system that facilitates the storage of all information on the performance scenario and its evolution.
5. Design of a secure and resilient network system interconnecting first responders, technological assistants, and the command post.

## Methodology and technologies

The INTREPID project work plan involves practitioners throughout the project using an iterative, user-centered methodology that evaluates the system regularly. Several pilots are organized using different scenarios (including natural disasters, industrial disasters, and malicious attacks in public spaces) to test the technology.

**Fig. 3:**
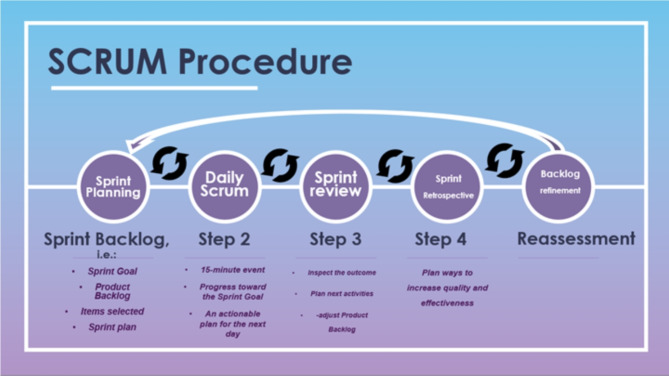
SCRUM performing structure

INTREPID has a work plan that involves end-users, from diverse backgrounds, and cultures and with different expertise, during all phases of the project to identify their needs and consideration constraints based on the evaluation of intermediate results, and their understanding of the possibilities and limitations of the technology. This is done as follows:

1. Iteratively revise the requirements and technical deliverables based on the results of the intermediate assessment.
2. Opens the project to end-users and advisory board and stakeholders outside the project.
3. Employs innovation management techniques, including co-creation sessions, to bridge cultural differences and organize productive interaction between early stakeholders and researchers.
4. Implements a SCRUM [22, 23] methodology for science and technology application.
5. SCRUM is a framework for developing, delivering, and maintaining products in a complex environment, with an initial emphasis on software development, although it has been used in other fields, such as other research projects [24] sales, marketing, and advanced technologies. The next Fig. 3 gives an overview of the SCRUM procedure.

The iterative user-centered methodology it proposes allows it to constantly consider and address end-user needs during system design and development to ensure optimal customization and maximize the chances of adoption of project results by professional users.

The field validation of the INTREPID tools follows a case study design [25] that compares the performance of the system with the expected results for each tool and pilot. To ensure that relevant aspects are measured and meaningful conclusions are drawn, the following steps were carried out [26]:

- An overall expected value was developed for each asset applied in emergencies.
- Planned the expected results of the use of the asset in a given disaster scenario.
- Developed scenarios with adequate performances to ensure that validation can be assessed.
- Conducted field data collection to ensure measurability of relevant parameters.
- Retrospective analysis of data to compare actual with expected results.

**Fig. 4:**
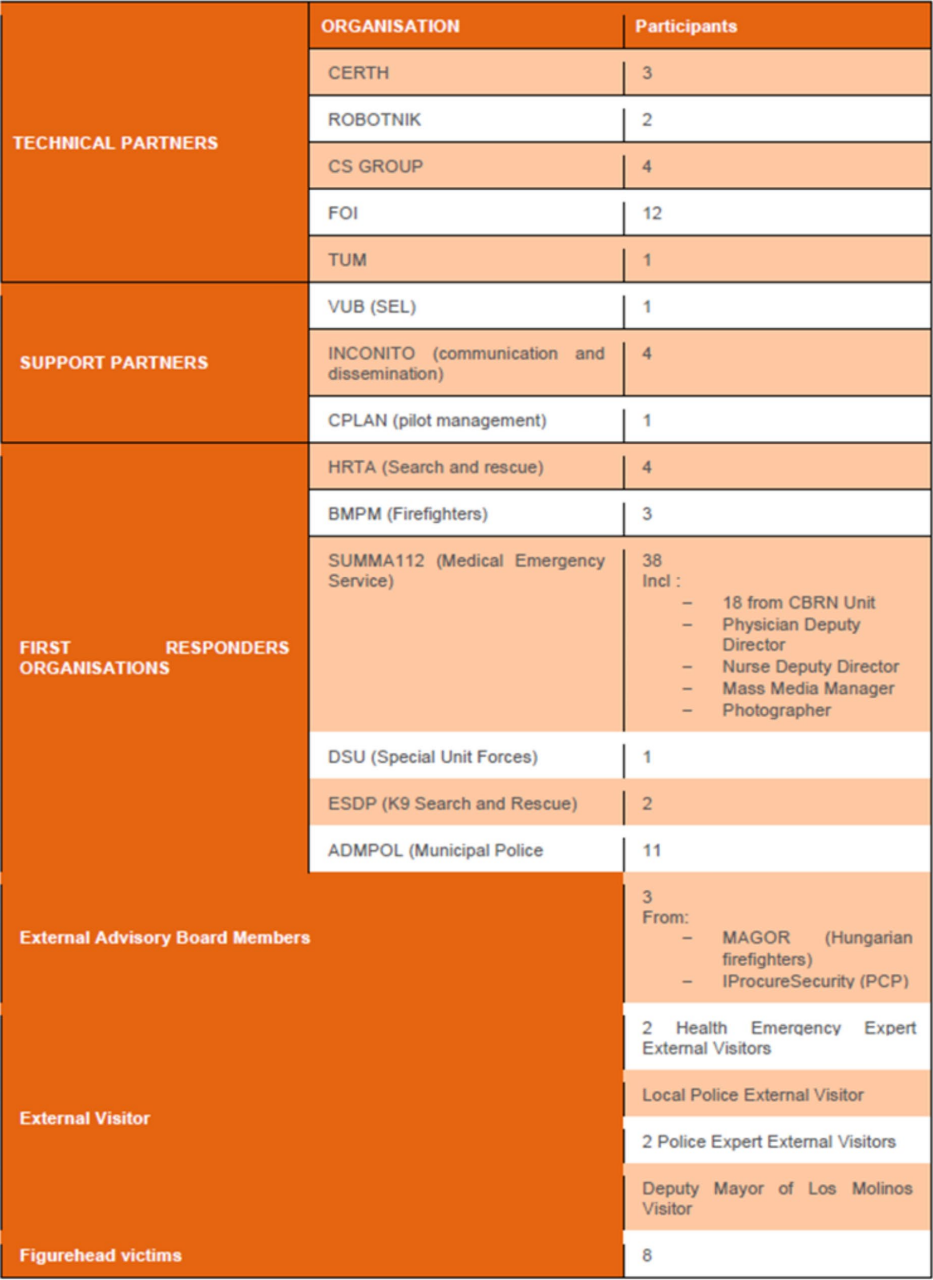
Graph of participants in the questionnaires

Periodic meetings were held to confirm the design and functionality of the different tools. Usability, efficiency and practicality questionnaires were designed to be filled before and after each pilot to confirm the development of the tools, which were aligned with the objectives of the first responders applied in the disaster field. These questionnaires are available in Appendix A. The feedback of the qualitative and quantitative questionnaires of the final pilots developed in Madrid, had the multidisciplinary participation of all the emergency end-users and technical partners of the project, as reflected in Fig. 4.

The INTREPID consortium pays due attention to the acceptability of the proposed solution by citizens and has dedicated a partner, a European expert in the field (VUB), to this end, considering social, ethical, and legal issues. So, another main task of the INTREPID project has been to identify and define the ethical and legal risks posed by the project activities and their outcomes and propose appropriate mitigation measures where necessary.

INTREPID combines the concept of Intelligence Amplification, Artificial Intelligence technologies, Extended Reality, as well as automated UXVs, innovative telecommunication, and positioning capabilities, to improve the rapid exploration and assessment of affected areas, determine the location of victims and threats and support the rescue operation, as well as the neutralization of dangerous sources, even in areas of difficult or dangerous access.

Paper prototyping and wireframing methods are used to stimulate discussion and involve the end-user in the design process.

Through a tailored impact assessment, INTREPID identifies and evaluates the potential impact of the system on European societal values and relevant legal and ethical requirements, with particular attention to safeguarding personal data and privacy.

- INTREPID Mobile System (INMOS) provides the command chain as well as first responders with improved situational awareness and operation management capabilities. It is the core system, based on Crimson technology that runs on ruggedized laptops, tablets, and smartphones, anywhere in the field or at a C2 Center.
- Digital Model Module: This central module of the INMOS stores all known information about the environment and situation, an out-of-core architecture available in CS’s VirtualGeo toolkit [27] that is used to implement this module.
- Extended Reality (XR) is the emerging concept that represents a working environment composed of a continuum of virtual, augmented, mixed, and real worlds. It is a novel approach that is implemented by this module to support visualization and interaction between devices with the Digital Mockup in 3D, Virtual, and Augmented Reality, using the HoloLens among others.
- Intelligence amplification module: This other module amplifies the collective intelligence of first responders to optimize the exploration of a site and the assessment of a situation involving multiple responders using multiple gas sensors and cyber helpers, the unmanned aerial and ground vehicles.
- Symbiotic operations control module: This last module integrates tools and algorithms to enable intelligent control and collaboration between cyber assistants.

The results of all these technologies tools proceed from the iteratively revised requirements and technical results from intermediate evaluations. These evaluations are provided by INTREPID stakeholders.

Three piloting and testing exercises have been conducted for this testing and evaluation:

- STOCKHOLM (SWEDEN): Subway flooding developed in November 2021.
- MARSEILLE (FRANCE): Explosion in an industrial estate organized for October 2022.
- MADRID (SPAIN): Hospital explosion. An intermediate pilot was carried out last March, the Los Molinos sanatorium has been used as the third pilot site. A chemical incident in a public building was simulated:

### Madrid pilot description

The final pilot was developed in Los Molinos, located north of Madrid, (Spain), on September, 20, 2023.

The organization and coordination of this pilot were carried out by the SUMMA 112 team (Medical Emergency Service of the Community of Madrid).

The site chosen for the execution of this exercise was the Los Molinos Sanatorium https://goo.gl/maps/SzBWBun3oun1TXfi9. The state of this building made it possible to simulate the conditions of an explosion: fallen walls, broken stairs, and collapse of internal structures. Victims were previously distributed in places that were difficult to access with communication difficulties, which brought the exercise closer to the real conditions that the first responders face with the technologies of the INTREPID project.

The exercise was deployed in several phases; in each one of them, the technologies used at each moment and the first responders who use them are explained.

- Phase 1:

Several calls are received on the 112 emergency telephone number reporting an incident with possible victims in a hospital. From these calls, it was gathered preliminary information of importance for the action:

- Fire/explosion in a hospital.
- Cloud of smoke.
- Estimation of the number of victims.

The emergency services were alerted by going to the location of the Municipal Police, a crew of firefighters and an advanced resource of SUMMA 112. It is considered to launch the INMOS platform for the management of the incident. After the initial assessment by the first advanced health resource that comes to the scene, activation is considered, according to the SUMMA 112 procedure of the Mass Casualty Incident (MCI) procedure, considered as any incident in which the assistance of at least ten victims, or in which there is a CBRN risk, given the magnitude of the event.

Upon checking the scope of the incident, and with the information provided by the SUMMA 112 Advance Life Support resource and taking into account the PLATERCAM (Emergency Plan of the Community of Madrid) and the SUMMA CBRN unit are activated. The initial response, managed from the CECOP (remote operations center) is in charge of the following teams (each led by a Commander):

1. Firefighters of Stockholm (Storstockholms brandförsvar) and Hellenic Rescue Team of Attica (Greece).
2. Medical Services (SUMMA 112).
3. Police of Madrid and Brussels.
4. Dog rescue Team.
5. SUMMA112 CBRN ( Unit with Chemical decontamination line).

The FR who arrived at the scene confirmed the details of the incident. It was informed to the police, fire services and SUMMA chief of the situation state (location of the explosion, possible risks, etc.). Based on this first assessment, the police commander sets up the command post, to the west of the building. The firefighters deploy the antenna so that the Command Post can make use of the INMOS. The FR secure the place to facilitate access of the necessary emergency services to the place.

b. Phase 2:

The FR that attended to the emergency calls (SUMMA112 Staff, Firefighters and Local Police) focused on what emergency professionals need during the pilot, based on the capacities of new technologies tools that could improve our work in the disaster.

A reconnaissance of the area was carried out, using UAVs and UGVs, identifying the perimeter of the incident focused on the delimitation of hot zones, and locating risks (location of toxic cloud) due to the storage of chemical substances inside the Hospital laboratory area.

With all the above information, the medical services decided on the strategic location of the Advanced Health Post and the decontamination unit, also considering the direction of the wind, shared instantaneously within the INMOS platform. The first aid personnel were adapted, according to the type of incident identified, with personal protective equipment, marking it in the INMOS platform.

Valuing the information provided by the INMOS platform, an initial estimate of the number of victims was made to adjust requests for resources and needs within the cyber assistants. The area was mapped with drones with sensors that provided information on the risks present and possible damage to the building, as well as the location of the victims.

The UAVs and UGVs also helped to locate viable entrances and exits and safer accesses to the building, as well as the bests secure entry to the victims (location and coordinates), where the danger of chemical substances in the environment, or the danger of deflagration were not marked by the sensors, thus confirming the safety of the teams involved.

The Real-Time Positioning Module (RTPM) provided us with the teams’ geolocation, shared through INMOS. It was visible the incident threats with the toxic cloud, so facilitated the action more quickly and in a targeted manner, increasing the security of the FR.

A preliminary visual triage was made, carried out by the UAVs and UGVs. The remote START triaje was made by SUMMA112 professionals (status and appearance of the victim in place), with the robot remotely initiating this initial assessment, providing first aid to the victims.

c. Phase 3:

It is confirmed with this assessment that the incident is not intentional, all this information was shared by INMOS directly.

The RTPM allowed the tracking of the triage procedures to report the contaminated victims, proceeding to the sectorization and mobilization of the SUMMA112 CBRN Unit. The access to the victims was carried out by the relevant professionals in the specific entries, depending on whether the area was contaminated or not, the access security, and the route that was determined by the INTREPID technology.

d. Phase 4:

SUMMA carried the rescue and health care of the different victims with the Advanced life support professionals. The information about victims’ states was shared in INMOS.

RTPM provided information instantaneously in real-time from the rescuers, the SUMMA emergency health workers, as well as the air and ground vehicles. The INMOS Platform improved the coordination between all the teams and operative and coordination staff.

**Fig. 5:**
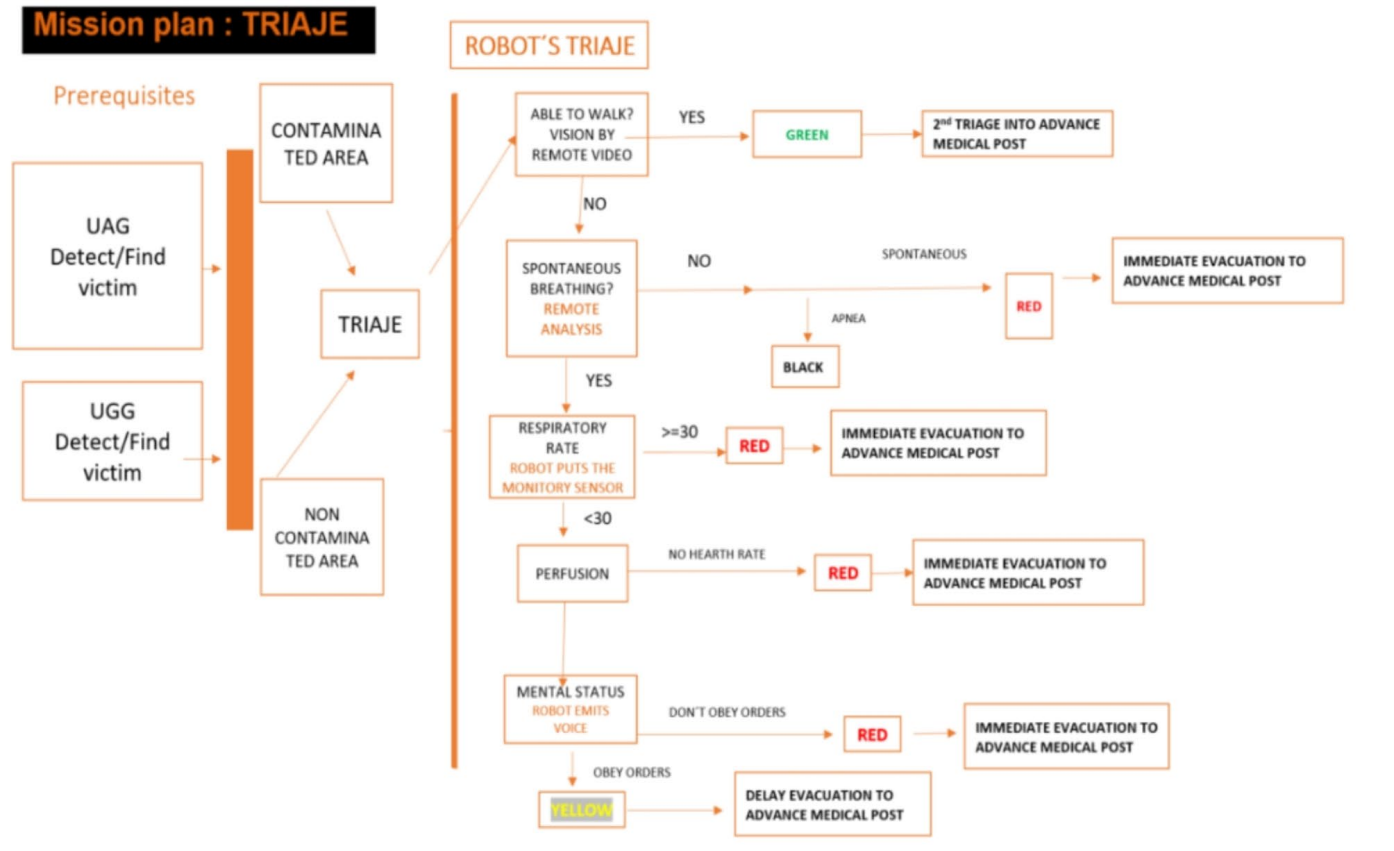
Robot’s Diagram performing the triaje
  * START (Simple Triage and Rapid Treatment) is a method for triaging victims in accidents and/or disasters with multiple wounds. The objective is to assess multiple victims in less than 60 s. It is a simple, fast, and highly sensitive method for detecting serious injuries [15].
  * Another goal is to localise faster the safe entrances and exits, and safer access to the building, as well as access to victims, where sensors do not indicate the danger of chemicals in the environment or danger of deflagration, detecting risks. It helps to maintain the emergency professionals’ health and security.
  * Areas of the building most likely to be exposed to a toxic cloud due to the storage of chemicals. The UAV will autonomously scan and generate 3D maps on the ground.

Achieved GOALS in the Madrid pilot:

- INMOS: Common platform usage by all stakeholders Task list - checklist for triage, for mission, and mobile-to-desktop management, and data visualization (image and video of UGV, UAV and risk areas).
- Route planning module: Allowed the visualisation of the trajectory within the terrain map using HoloLens.
- Environment assessment module: Location tracker and duplicate avoidance.
- Environment mapping module: faster gap detection and alignment, faster and with UGB and UAV.
- Real-time positioning module: Display of peers both with RTPM and INMOS devices.
- UGV: Autonomous area scan testing with localization and triage.
- Symbiotic operational control module: improved the communications between all stakeholders involved, optimal area division, and mission status reporting.
- Network connection: Testing in the pilot building to cover larger areas, no disconnections, good speeds, and coping with indoor complexity. It favored the cyber-assistants would share the Information on the INMOS platform. It was distributed between all the first responders’ pilot, carried out so far:
  * Drone mapping with sensors that will provide information on risks and possible damage to the building and location of victims.
  * Risk detection/assessment following the explosion in the storage area of these substances inside the Molino´s Hospital.
  * Visual information from Robots (UGV) scanning the building for the detection of victims and ruling out the presence of possible aggressors.
  * UGVs provided a breakthrough by equipping the platform with four folding robotic legs on wheels, which allow for climbing stairs and overcoming obstacles. It also was added an articulated arm with a highly sensitive 3-finger gripper adapted to open doors, turn lights on and off, control elevators, and place monitoring sensors on detected victims, along with a two-way audio/microphone and video technology that allows the cyber-assistant to develop a START first triage without putting the SUMMA staff at risk (see Fig. 5).Through the smart wristband, the heart rate, blood pressure and saturation sensors provide sufficient basic data for the start triage and thus we screen the patient and assess whether he is alive, whether he is breathing or not breathing and whether he has a radial pulse.

The INTREPID (INMOS) system had suggested the quickest and safest access routes.

Both UAVs were designed to absorb heavy impacts, evolve in populated areas and avoid injuries in case of a crash thanks to their foam frames. Their innovative payload plate was modular to allow for quick plug-and-play of sensors.

* Detection/assessment of the risk after the explosion in the storage area of these substances within the Navacerrada Hospital.
* Visual information from Robots, scanning the building for victim detection and discarding the presence of potential aggressors.
* Mapping with drones with sensors that will give information on risks and possible damage to the building and location of victims.
* Viable entrances and exits, and safer access to the building, as well as to access the victims, in which the sensors do not mark danger of chemical substances in the environment or danger of deflagration. -
* Areas of the building, with a higher probability of exposition of a toxic cloud, due to the storage of chemical substances. The UAV will be to scan autonomously and generate 3D maps on the ground.

Both the fastest and most secure access routes had been suggested by the INTREPID system (INMOS).

Both unmanned aerial vehicles were designed to absorb heavy impacts, evolve in populated areas, and avoid injuries in the event of a crash thanks to their foam frames. Its innovative payload board was modular to allow for fast plug-and-play of sensors.

In this case study, a triangulation was developed to examine the approach and applicability of the different INTREPID tools from the priorities of the main emergency services involved in CBRN Search and Rescue operations: Health emergency personnel, Professional firefighting search and rescue teams, and Law enforcement.

It was developed a semy estructured questionnaire to be filled by the diferent emergency services that particpated in the pilots to be filled pre and post pilot.

With the responses obtained in the post-test and in the focus group of the participants in the emergency health care pilot, the answers were structured according to the SWOT [28] analysis. A SWOT analysis (or SWOT matrix) is a strategic planning and strategic management technique used to help individuals or organisations identify strengths, weaknesses, opportunities and threats for business performance and/or project planning.

## Results

The deployment of INTREPID visible tools (INMOS, RTPM, UAV, and UGV), the other applied tools (XR, IA, etc.) and the actual execution of these in pilots, with end users, have been demonstrated, applied, and evaluated by measuring their quality and usability in collaboration with technical partners; focusing on how first responders use the developed technologies in real-world pilots.

For this reason, before and after the development of each scenario, where all INTREPID tools have been tested, the qualitative and quantitative evaluation of the same was implemented.

After working with members of FOI (Swedish Defence Research Agency); that developed the Field Validation Methodology, a qualitative questionnaire was shared to the first responders with questions such as: Did we achieve faster exploration thanks to the use of these tools? Could we achieve a higher quality and quantity of victims saved? Do we work in safer conditions? Is it necessary to count with personnel trained in these technologies? Do they require less personnel? How could the use of each technology be improved?

**Fig. 6:**
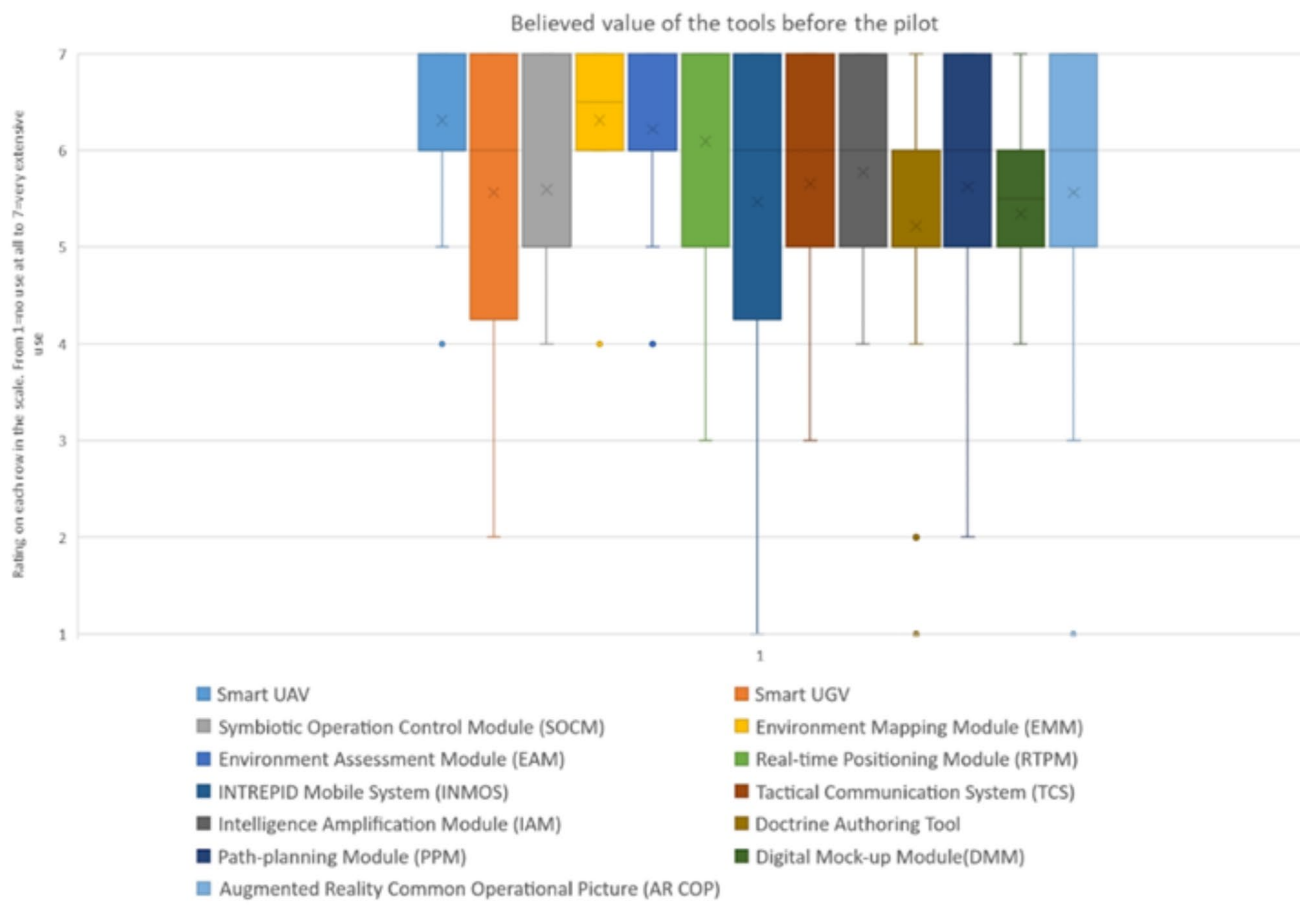
The end users rated believed value of each tool before the scenario on the scale 1-7 ( 1=no value at all – 7=very extensive value)

**Fig. 7:**
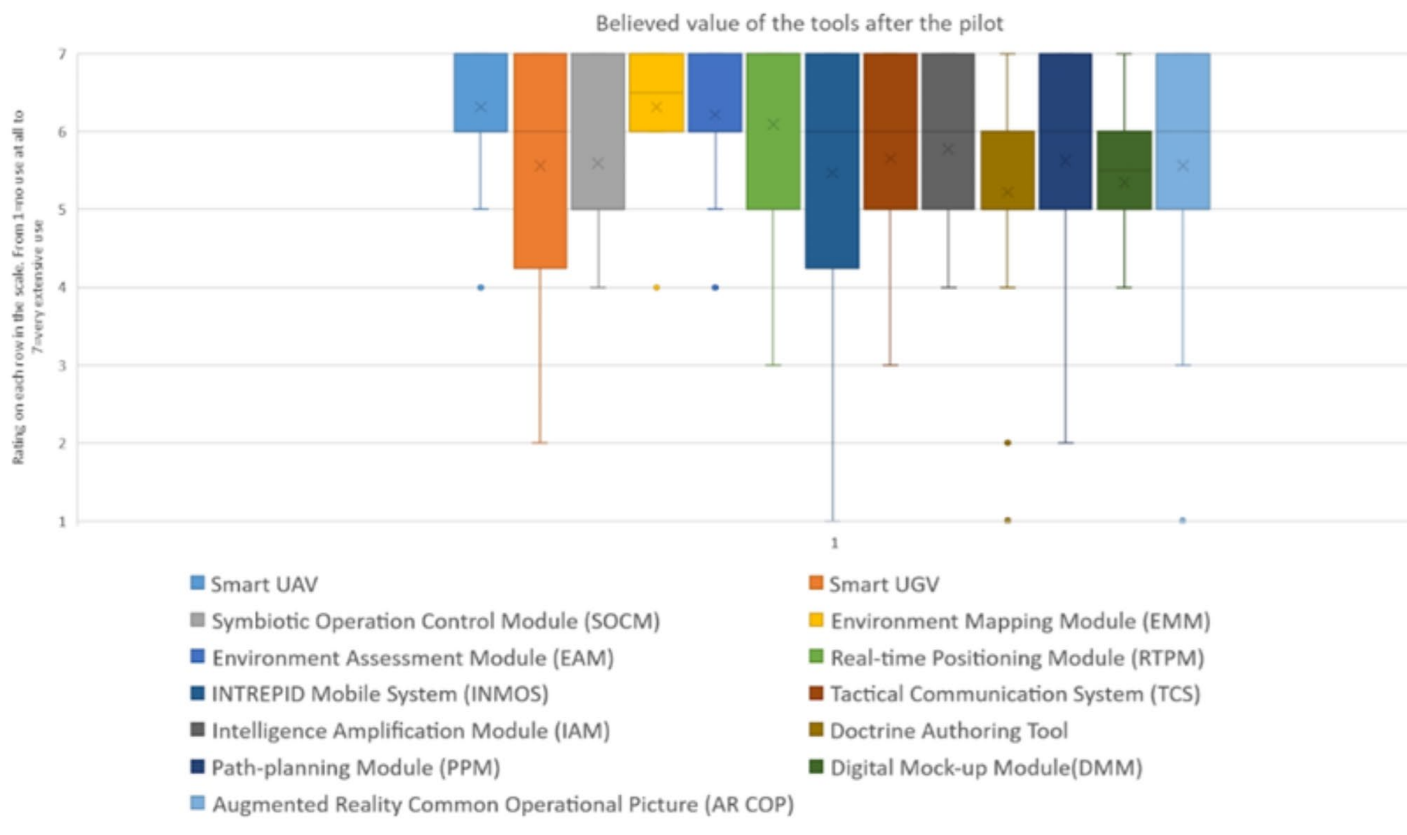
The end users rated believed value of each tool after the scenario on the scale 1-7 ( 1=no value at all – 7=very extensive value)

The questions related to the expected value were intended to go beyond what the tool does, seeking to describe the aspects that benefit the first response missions of the tool in question and what weaknesses are detected in order to be able to implement the necessary improvements (Figs 6 and 7).

It has been developed SWOT Analysis for assessing internal and external factors, as well as the current and future potential of the INTREPID tools:

### Strengths

Technological tools integrated into incident support procedures increase safety, coordination, and communication between the teams involved, enhancing the resilience and efficiency of healthcare while reducing risks. When we compare it with the tools that are currently used, it is when we see the improvements that these new technologies produce.

**Fig. 8:**
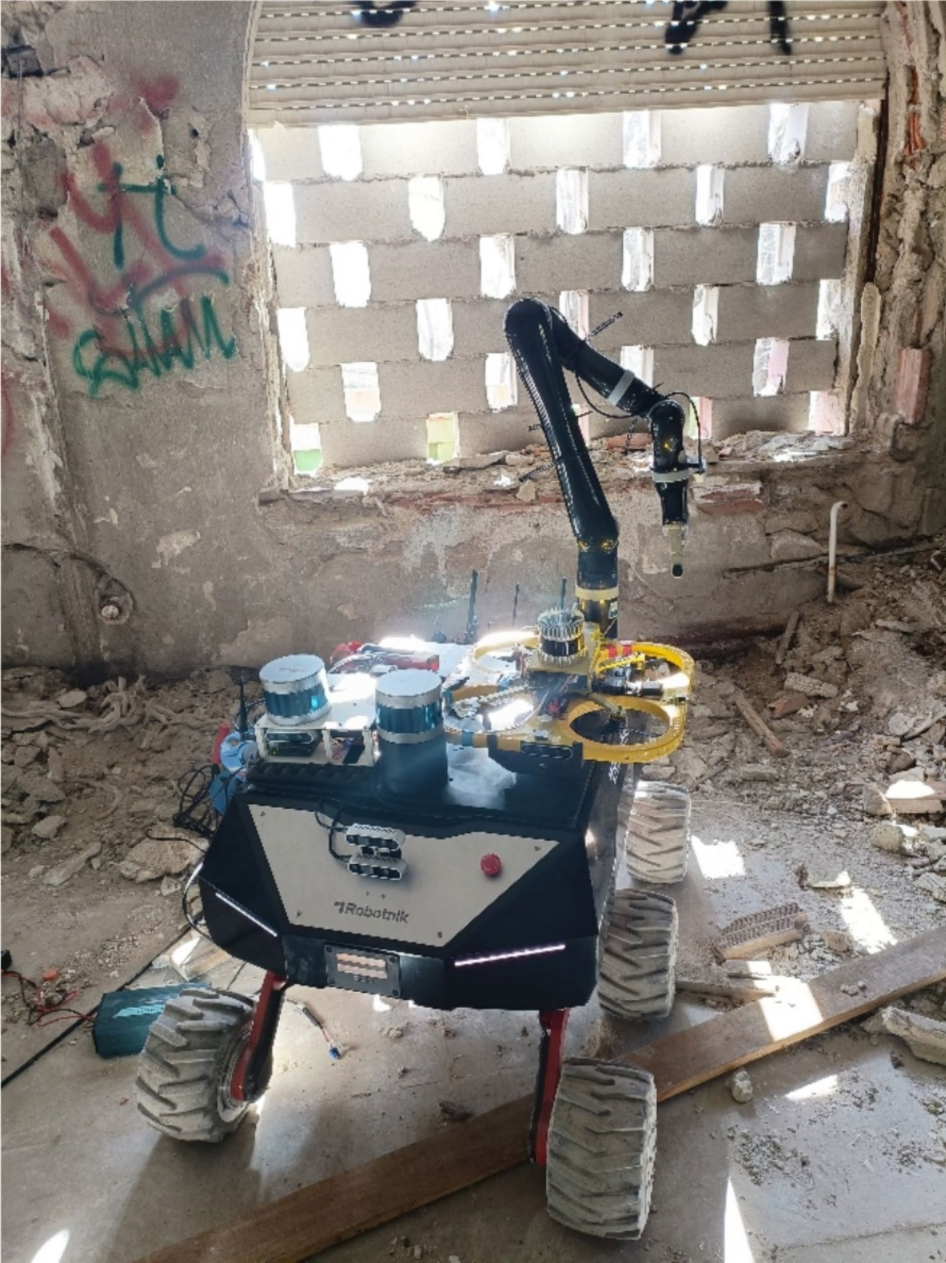
Madrid pilot, an image of a robot and drone triaging in the chemical incident

Following the 3 pilot in Madrid (see Fig. 8), INTREPID has shown to be a platform providing effective intergroup communication, real-time information gathering, and coordination through technology adapted to our needs, helping to reduce the initial chaos of disasters, thus improving the teamwork of first responders. Currently, communications are made by the Tetra system (Trans European Trunked Radio). Most of the countries in the world have reserved a band for critical communications that is usually around 380–400 MHz and by using such a low frequency it allows greater coverage to be achieved for each of the installed antennas. The independence of the usual network of the new Crisom technology allows communications to be maintained in case it is damaged or absent, an important advantage in this type of incident.

It should be borne in mind that the positioning, which is highly challenging within indoor environments, so that it can be maintained over time, or the synchronization of data from all technologies, are priorities called Super mission. The data are collected in a proprietary data cloud, not an external one, with absolute priority being given to the protection of the data received. Another essential factor is that the application allows artificial intelligence to be applied to specific objectives that are a priority in disasters.

Another fundamental concept in disasters is the TRIAGE, the system marks the identification of victims with standardized categorization, according to the most widely used international triage procedures, to facilitate that the information obtained on the victims (outputs) of the cyber-assistants allows the immediate coordination of the first responders according to their seriousness and risk. The UGV has managed to perform the first triage (using the START method [29]), so that the health professionals have triaged the victim, avoiding exposure to the toxic substance.

We are talking about saving a lot of time by being able to start that triage remotely. In other circumstances, it would be necessary to wait to secure the area and be able to reach the victims, so that with this new technology time and effectiveness are gained, improving the assistance and survival of the victims.

#### Augmented Reality Module (virtual reality glasses)

This module allows a remote user, either in the field or in a remote location, to assist another first responder equipped with AR/MR glasses. The remote user will be able to see what the first responder sees, captured by their glasses, and send visual annotations that will be displayed on the glasses. This module also allows the user to take data about the environment, leaving virtual messages, indications, and alerts of its position that others can see on the 3D map or through an AR device.

Once again, we managed to reduce action times and we can apply saving measures more quickly, always with medical support.

#### Route planning module

This module provides navigation assistance to participants and cyber assistants to enter or leave an area. It will take advantage of the digital mock-up of the site, fed with data captured on the ground, including reconstructed parts of the terrain, together with dynamic situation information such as the location of emergency units. In addition to helping the user in their exploration of the environment, it will allow the first responders to control the UGV and UAV using high-level orders such as “go there”, “leave now,” or “scan this area”, improving security.

### Weaknesses

In a chemical hazard incident, the toxic cloud that forms can affect the walls of both drones and robots, which are the cyber assistants that in the first instance provide information on risks and the location of victims within the disaster area.

It is necessary to confirm the protection covers against this chemical risk to avoid corrosion and to confirm the functionality of the cyber assistants in toxic environments.

### Opportunities

Two types of UAVs have been designed, the first will be adapted to the rapid exploration of large areas not very obstructed, while the second will adapt to the exploration of disordered interior environments of several floors, even using elevators (detecting buttons, reading floor numbers, pushing buttons) or turning on lights.

Both unmanned aerial vehicles and unmanned ground vehicles are designed to absorb heavy impacts, evolve in populated areas, and avoid injuries in the event of a crash thanks to their foam frames. Its innovative payload board is modular to allow for fast plug-and-play of sensors.

Another feature of the UAV is to scan autonomously and generate 3D maps on the ground.

The tools that are being generated by this project, both UGV and UAV improve the efficiency and safeguard of first responders when assessing an area where a catastrophe has occurred. These tools are going to help to save assessment time, which facilitates a faster and safer rescue of victims. These tools have been adapted to the needs of the first responders.

The INMOS system connects to the INTREPID network and receives all the information collected by the sensors, the position of the units, as well as any support request, report, or command that is intended for the user. Thanks to its open and distributed design, several interconnected INMOS can be used for collaborative sessions with multiple users sharing the same situational information (with proper access rights management) with Push-To-Talk capabilities and remote assistance.

The improvement of the transmission of this data, which is normally done by Tetra or telephone, is due to speed and data quality.

### Threats

Radio data transmission in use in most European countries: in man-made incidents, is usually avoiding any transmission network available by terrorists so that they cannot firebombs and cannot communicate with other terrorists; as a result, it is recommended only to use “preserved” frequencies and should avoid using public frequencies (public operators immediately stop any public network in the area of a disaster).

Coverage versus data rate: to achieve a high data rate, it is important to get good radio coverage - the only way in a building is to multiply the radio infrastructure points inside the building; unfortunately, that means establishing links between these points and the central equipment - which is not possible with cables, and quiet impossible with radio links (especially in case of non-specialized first responders and/or especially in case of jamming); that is why it is proposed to get a single central site (in general outdoors) with enhanced sensitivity - which is the proposed way for any “tactical” radio network. The datasets generated and analysed during the current study are available in the INTREPID repository, https://intrepid-project.eu/public-results.

## Discussion

It can be found in many articles where UAVs are used in transporting materials and exploring areas [5, 30–32], but not using UAVs and UGVs collaborating in dangerous areas is too risky for first responders, and even less so if we apply it to out-of-hospital health emergencies.

Both UAVs and UGVs can quickly transport materials for emergency treatment, and infrared cameras for locating people both day and night due to the ability to detect body heat. The possibility that these tools scan the area, detect and locate casualties, being located on a map with a minimum margin of error, makes this type of project demonstrate its usefulness and, by demonstrating its effectiveness, we can promote these improvements in interventions.

All this speed and improved response, risk detection, and increased security in the area produce an improvement in the organization of the incident, being able to develop standard security procedures for different scenarios. In addition, being able to continuously improve the tools means that they can be adapted to the different situations that are created.

The INTREPID project began to develop during the COVID19 pandemic, which has significantly affected face-to-face meetings, which are important in this type of project. Telematic meetings have replaced face-to-face meetings, and this problem has been partially resolved. Another limitation found in the little bibliography was no examples of UAVs and UGVs in catastrophes and out-of-hospital actions.

The information that we collect from the UAVs and UGVs in the pilots when we complete the INTREPID project will be able to confirm that they are an essential resource in disaster assistance since they facilitate the inspection of the affected area, help in the search and location of possible casualties, bring critical resources where other means are not able to reach, they provide greater security since they operate in risk areas for FR, they detect danger zones in unstable collapsed structures, as well as possible toxic substances, giving information to FR on the most appropriate measures to take to approach the area, and what measures First Responders (FR) can take to treat and decontaminate the victims who will be evacuated. In this way, the action of the FR is optimized.

The usability ratings of the tools before and after the final pilot remain in quite similar proportions, understanding that the state of the tools are in a Technology readiness levels (TRL) [33] of 7–8. Its a balance between the usability and efficiency of the tools and the maturity of them.

This study has some limitations. We did not have access to all health experts in the field of new technologies applied in hazardous search and rescue operations, so we could not gather their opinions. Another limitation of this study is that different views may apply to the classification of the elements in the four SWOT categories, and one opportunity may be considered a strength by another person. Likewise, one person may see an element as a threat and another as an opportunity. In this study, an attempt has been made to consider the most common views in order to reach a consensus.

It should be noted that none of the people involved in the focus groups and questionnaires have a commercial or personal relationship with the technical partners who are the developers and manufacturers of the tools.

## Conclusions

The project highlighted the need to improve the coordination for controlling and organizing what is happening on the ground during an emergency through more effective communication between first responders and/or with the operational control centers.

The INTREPID project has supported this need by providing tools to be used in emergencies, especially in the first moments of disasters when uncertainty and consideration slow down rescue response and the area is often not secured enough for first responders to intervene.

The time that can be gained from adversity is decisive when coordinating emergency teams and speeding up the search for victims in critical areas, as is the detection of risks before they can cause casualties among the first responders. In a disaster, any type of risk can arise, since the destruction, such as that caused by an explosion, can affect chemical installations and gas pipelines, or even favor spills of hazardous materials usually controlled by man.

INTREPID technology with Crimson software, a ground-breaking and widely used system for crisis management, the INMOS platform, UGVs, and UAGs helps to control and counteract these risks, just as technology has helped in other specific situations so that these tools have ceased to be a theoretical material against CBRN risks, to be used and valued fighting in reality against these risks.

The immediate need for communication and coordination in the face of the initial chaos of the emergency has been demonstrated and is being covered, gaining time on favors of the assistance to the victims in these types of incidents both produced by man as the explosion in Spain, or the dumping in France and natural as the simulated flood in Sweden.

This project has received funding from the European Union’s Horizon 2020 research and innovation program under grant agreement No. 883,345. The authors thank the European Commission for financial support of the INTREPID Project.

## Electronic supplementary material

Supplementary Material 1.

## Figures and Tables

**Fig. 9:**
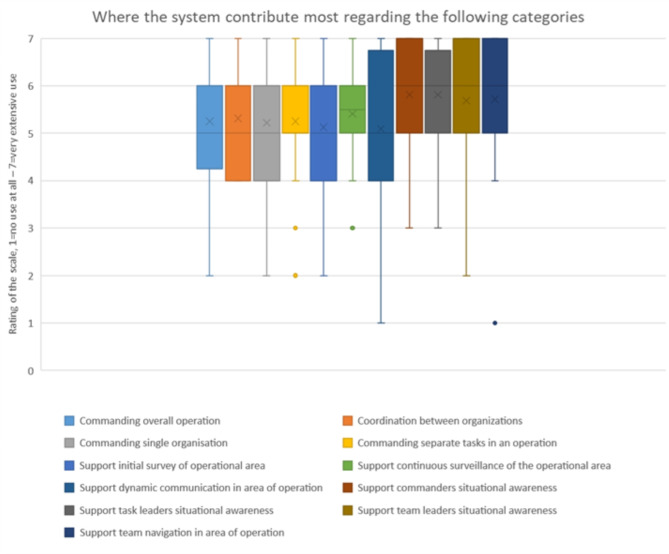
Ratings of the usefulness of tools in intra-organisational, co-ordination and community search and rescue operations(*N*=28). Ratings on scale 1= no use at all to 7= very extensive. Box= lower and upper quartile, x= mean value, line inside box= median, whiskers= minimum and maximum, dots indicate outliers

## Data Availability

The Data Availability Statement is provided on this link, https://intrepid-project.eu/public-results.

## Appendix A. Pre and Post Pilot Questionnaire

The original surveys are available this link, https://goo.su/MlPuc.

The results of the qualitative assessment by the emergency care participants in the testing of the tools extracted from the anonymous questionnaires.

It was confirmed that the experience of emergency end-users was sufficiently valid to be adecuate candidates to this case study with question 1 of the questionnaire.

## Appendix B. Summary of the most representative results of INTREPID tools evaluation

The first item of the questionnaire prepilot (own Experience) served to confirm that the experience of emergency end-users was sufficiently valid to make them suitable candidates for this case study.

The second item values the operational procedures necessary for the effective deployment of emergency services in disaster areas; respondents rated as most useful the support provided by INTREPID tools, initial assessment of the disaster area, continuous monitoring, dynamic field communication and awareness of the disaster area (Fig. 9).

Questions 3 and 4 reflects  result in the threat part of the SWOT analysis.

The responses among the participating members, concerning to the value of the use of the tools, before and after the simulations or scenarios was of high value, due to the benefits of their use, above the difficulties of handling or weaknesses of the technologies.

**INTREPID Mobile System (INMOS)**

- ***In your opinion, what is the expected value of this tool for your profession?***

The expected value of this tool is to improve inter coordination between first responders and cybernetic assistants, improving communication between them by being connected, providing important information such as risk location, safe access, where the team is at all times, location of victims, reporting a global view of the incident.

b) ***In your opinion, what modifications are needed to reach a satisfactory level of usefulness?***

For the tool to work in optimal conditions, it is necessary to achieve optimal coverage at the scene of the incident, train and educate the teams about the mobile system before pilots and real scenarios to obtain the best results with the tool.

c) ***Did you interact with the tool during Pilot 3 and if so - how?***

In general, all the participants’ pilot handled the INMOS platform to a greater or lesser extent. The general evaluation was that it would be desirable an advanced training for getting the best efficiency in the results.

d) ***How would you describe the decision-support offered by INMOS, compared to what you would normally have used?***

Without this technology, when a mass-casualty incident occurs, communications are conducted only by radio voice; and medical care teams, in the incident area do not have access to existing risk information until law enforcement, security, and fire departments communicate it to their superiors, and this information is shared in remote coordination or in person if we are close to the area; with delayed response times.

In these cases, communications are more difficult, which is why the Intrepid Mobile System (INMOS) is very useful in improving the effectiveness of first responders by optimizing operational time and security coordination on the scene. Mostly when risks can affects to the population and first responders of the area.

**Real-time Positioning Module (RTPM)**

- ***In your opinion, what is the expected value of this tool for your profession?***

The expected value of the Real-Time Positioning Module is to know first-hand, at all times, the geolocation of the professionals who are intervening at the scene and, in addition, of the victims.

b) ***In your opinion, what modifications are needed to reach a satisfactory level of usefulness?***

Perhaps one of the greatest weaknesses of this tool is sometimes the lack of good coverage. The difficulties in connectivity and adequate coverage mean a delay in the reception of data from the intervention area.

c) ***Did you interact with the tool during Pilot 3, and if so - how?***

SUMMA 112 members, firefighters and police were able to use and see the RTPM. The results could be seen through the INMOS Platform and were checking the usability in situ.

d) ***How would you describe the benefits offered by the RTPM (if any) compared to positioning equipment or methods you would normally use in similar situations?***

Under normal conditions, without RTPM in disasters, when professionals enter buildings or dangerous scenarios, there is a risk of losing the signal and it’s positioning. At that point, among first responders, the location of each piece of equipment would be unknown. Therefore, the RTPM is a major breakthrough, as both first responders and victims will be geolocated at all times in real time.

d) ***Based on experience from similar situations, how often would using the RTPM increase the feeling of safety of first responders?***

The general opinion is that the use of RTPM will increase the feeling of security in any of the situations or scenarios in which this tool is applied.

**Smart UAV**

- ***In your opinion, what is the expected value of this tool for your profession?***

The expected value of the use of drones is getting the information overview of the whole area in a short time, increasing the level of security, thanks to the shortening of scanning times of the area digitally by dangerous places, being able to the area and search for victims without risk, compared to the ordinary or usual way, which would be in situ, walking through the places at risk.

b) ***In your opinion, what modifications are needed to reach a satisfactory level of usefulness?***

An added value for the optimization of this tool is that it could be managed by any first responder, which would entail certificated training and learning prior to its use.

c) ***Did you interact with the tool during Pilot 3 and if so - how?***

This tool was mostly used by technical partners and security professionals. We were able to see its applicability, use, and transfer of images.

d) ***Based on your experience, how often would you deploy and use the Smart UAV in similar scenarios?***

The general opinion is that it would be of great utility and applicability to the deployment of drones in any of the scenarios or situations of catastrophes, for the infinity of advantages in their use of hazardous environment knowledge.

**Smart UGV**

- ***In your opinion, what is the expected value of this tool for your profession?***

Robots are of great value to us as professionals, as we are increasingly encountering dangerous multiple casualty incidents as chemical and/or biological scenarios or in environments where threats are caused by humans. The use of robots in these types of hazmat scenarios would improve the access to the victims.

Furthermore, the robots, upon arrival, can report a first assessment of the victim’s and can make a first triage, through a brief monitoring by the use of vital signs sensors, without risk to the first responders.

b) ***In your opinion, what modifications are needed to reach a satisfactory level of usefulness?***

One way to improve the use of robots would be to add a tracking sensor for the victims, added to the cameras with microphones so that there is a remote and constant visualization and communication with them.

c) ***Did you interact with the tool during Pilot 3, and if so - how?***

We were able to use the robot to do the first START triage in a chemical environment., being able to communicate and interview a victim, monitoring the basic constants of the patient.

d) ***Based on your experience, how often would you deploy and use the Smart UGV in similar scenarios?***

The entire SUMMA 112 team believes that the applicability and deployment of the robots would be useful and enriching in any type of CBRN scenarios performance.

Firefighters appreciate the usefulness of scanning chemical risk zones with the robot to confirm safe access zones and areas where safe personal protective equipment is needed. Urban Search and Rescue team and police also take in consideration the added value of robot scanning in potentially explosive areas.

There are many tools provided by the Intrepid Project technology, but it is worth highlighting that the most valued in helping in dangerous incidents or catastrophes are the Intrepid Mobile System Platform (INMOS), the Real Time Positioning Module (RTPM) and the cyber-assistants, Drones (UAV) and Intelligent Robots (UGV), being necessary the others for a proper and more complete functioning of INMOS and a global vision of the incident and its risks.

The INTREPID tools are very useful for advancing disaster coordination, improving, among other things, communication between teams, reducing risks such as human threats and other relationships with CBRN environments by first responders.

The information and tasks developed jointly by the cyber-assistants, shared by INMOS and geopositioning acquire a very important value for professionals when they are faced with a massive disaster with dangers in the environment and the possible threats that may arise from such scenarios.

When working in an interdisciplinary manner, interoperability and data protection is fundamental to avoid cyber risks, being one of the most important keys, as well as the geolocation of the professionals to correctly access the scenarios and therefore the victim.

The INTREPID tools are very useful for advancing disaster coordination, improving, among other things, communication between teams, reducing risks such as human threats and other relationships with CBRN environments by first responders.

The tools tested have identified ways to integrate research, including operational and applied research, into disaster preparedness.

It has demonstrated innovative processes and interventions to strengthen health systems to effectively prepare for, manage and respond to disasters, crises and emergencies with support for better recovering from emergencies and crises in environmental hazards without victims added.

This platform and the ICT tools integrated propose proven models for managing health emergency systems in crisis situations.
