# Supplementary material for: INtelligent toolkit for reconnaissance, assessments and prehospital support in Perilous InciDents: a realistic experiment in prehospital environment
Source: BMC Health Serv Res. 2024 Nov 1;24:1331. doi: 10.1186/s12913-024-11786-3 (PMC11529029; doi:10.1186/s12913-024-11786-3)
Supplement: Supplementary file 1 — Supplementary Material 1. [file 12913_2024_11786_MOESM1_ESM.docx]

# **Part 1 – BEFORE Pilot 3 test**

Alias: _________________________________
(Please choose an alias to be used for all surveys – do NOT use your name)

How long have you been working as a First Responder: ____________ years

With what profession:

- Fire fighting
- Law enforcement
- Urban Search and Rescue
- Medical/ Healthcare
- Other: _________________________

**Own Experience**

1. How do you rate your personal experience or ability concerning the tasks below?

Mark your rating on each row in the scale, 1=very limited – 7=very extensive.

| Very limited | 1 | 2 | 3 | 4 | 5 | 6 | 7 | Very extensive |
| --- | --- | --- | --- | --- | --- | --- | --- | --- |
| Working in large scale emergency response operations? | O | O | O | O | O | O | O |  |
| Collaborate with other organisations in emergency response operations? | O | O | O | O | O | O | O |  |
| Commanding large scale emergency response operations? | O | O | O | O | O | O | O |  |
| Using computerised command and control systems? | O | O | O | O | O | O | O |  |
| Execute command and control without computerised systems? | O | O | O | O | O | O | O |  |
| Execute command and control with computerised systems? | O | O | O | O | O | O | O |  |
| Using UAVs in emergency response operations? | O | O | O | O | O | O | O |  |
| Operating UAVs in emergency response operations? | O | O | O | O | O | O | O |  |
| Using UGVs in emergency response operations? | O | O | O | O | O | O | O |  |
| Operating UGVs in emergency response operations? | O | O | O | O | O | O | O |  |

**Your view on computerised support tools**

1. Where do you believe that the INTREPID tools will contribute the most regarding the following categories? Mark your rating on each row in the scale, 1=no use at all – 7=very extensive use.

| No use at all | 1 | 2 | 3 | 4 | 5 | 6 | 7 | Very extensive use |
| --- | --- | --- | --- | --- | --- | --- | --- | --- |
| Commanding overall operation | O | O | O | O | O | O | O |  |
| Coordination between organizations | O | O | O | O | O | O | O |  |
| Commanding single organisation | O | O | O | O | O | O | O |  |
| Commanding separate tasks in an operation | O | O | O | O | O | O | O |  |
| Support initial survey of operational area | O | O | O | O | O | O | O |  |
| Support continuous surveillance of the operational area | O | O | O | O | O | O | O |  |
| Support dynamic communication in area of operation | O | O | O | O | O | O | O |  |
| Support commanders situational awareness | O | O | O | O | O | O | O |  |
| Support task leaders situational awareness | O | O | O | O | O | O | O |  |
| Support team leaders situational awareness | O | O | O | O | O | O | O |  |
| Support team navigation in area of operation | O | O | O | O | O | O | O |  |

Comments on use and operational value: __________________________________________

____________________________________________________________________________

____________________________________________________________________________

____________________________________________________________________________

1. How big change do you believe such support tools would encompass for your organisation regarding methods, command and control, communications and behaviour?

Very limited 1 2 3 4 5 6 7 Very extensive

Comments on change: _____________________________________________________

____________________________________________________________________________

____________________________________________________________________________

1. What problems/risks would a system like this possibly mean for your organisation, regarding categories below? Mark your rating 1-7 on each row of the scale.

| No problems/risks | 1 | 2 | 3 | 4 | 5 | 6 | 7 | Extensive problems/risks |
| --- | --- | --- | --- | --- | --- | --- | --- | --- |
| Security for external persons getting access to the system | O | O | O | O | O | O | O |  |
| Time required for handling material, charging batteries etc. | O | O | O | O | O | O | O |  |
| System failure, for example on positioning units and objects | O | O | O | O | O | O | O |  |
| Problems due to delayed data communication | O | O | O | O | O | O | O |  |
| Limiting situational awareness because operators focus to much on the system | O | O | O | O | O | O | O |  |
| Problems with compatibility with other equipment and systems | O | O | O | O | O | O | O |  |
| *Own suggestion:* | O | O | O | O | O | O | O |  |
| *Own suggestion:* | O | O | O | O | O | O | O |  |

Comments on problems/risks: ______________________________________________________

_______________________________________________________________________________

**Your view on expected value of the INTREPID tools**

1. What is your *believed* value of the tool?
   Mark your rating on each row in the scale. (1=no value at all – 7=very extensive value

| No value at all | 1 | 2 | 3 | 4 | 5 | 6 | 7 | Very extensive value | I have no knowledge about the tool |
| --- | --- | --- | --- | --- | --- | --- | --- | --- | --- |
| Smart UAV | O | O | O | O | O | O | O |  | O |
| Smart UGV | O | O | O | O | O | O | O |  | O |
| Symbiotic Operation Control Module (SOCM) | O | O | O | O | O | O | O |  | O |
| Environment Mapping Module (EMM) | O | O | O | O | O | O | O |  | O |
| Environment Assessment Module (EAM) | O | O | O | O | O | O | O |  | O |
| Real-time Positioning Module (RTPM) | O | O | O | O | O | O | O |  | O |
| INTREPID Mobile System (INMOS) | O | O | O | O | O | O | O |  | O |
| Tactical Communication System (TCS) | O | O | O | O | O | O | O |  | O |
| Intelligence Amplification Module (IAM) | O | O | O | O | O | O | O |  | O |
| Doctrine Authoring Tool | O | O | O | O | O | O | O |  | O |
| Path-planning Module (PPM) | O | O | O | O | O | O | O |  | O |
| Digital Mock-up Module(DMM) | O | O | O | O | O | O | O |  | O |
| Augmented Reality Common Operational Picture (AR COP) | O | O | O | O | O | O | O |  | O |

# **Part 2 – AFTER Pilot 3 test**

Alias: _________________________________ (the same alias is to be used for all surveys,
do NOT use your name)

**Questions about the INTREPID Tools**

1. What is your *believed* value of the tool after the scenario?
   Mark your rating on each row in the scale. (1=no value at all – 7=very extensive value

| No value at all | 1 | 2 | 3 | 4 | 5 | 6 | 7 | Very extensive value | I have no knowledge about the tool |
| --- | --- | --- | --- | --- | --- | --- | --- | --- | --- |
| Smart UAV | O | O | O | O | O | O | O |  | O |
| Smart UGV | O | O | O | O | O | O | O |  | O |
| Symbiotic Operation Control Module (SOCM) | O | O | O | O | O | O | O |  | O |
| Environment Mapping Module (EMM) | O | O | O | O | O | O | O |  | O |
| Environment Assessment Module (EAM) | O | O | O | O | O | O | O |  | O |
| Real-time Positioning Module (RTPM) | O | O | O | O | O | O | O |  | O |
| INTREPID Mobile System (INMOS) | O | O | O | O | O | O | O |  | O |
| Tactical Communication System (TCS) | O | O | O | O | O | O | O |  | O |
| Intelligence Amplification Module (IAM) | O | O | O | O | O | O | O |  | O |
| Doctrine Authoring Tool (DAT) | O | O | O | O | O | O | O |  | O |
| Path-planning Module (PPM) | O | O | O | O | O | O | O |  | O |
| Digital Mock-up Module (DMM) | O | O | O | O | O | O | O |  | O |
| Augmented Reality Common Operational Picture (AR COP) | O | O | O | O | O | O | O |  | O |

**

**

1. **INTREPID Mobile System (INMOS)**
2. In your opinion, what is the expected value of this tool for your profession?

__________________________________________________________________________

__________________________________________________________________________

1. In your opinion, what modifications are needed to reach satisfactory level of usefulness?

__________________________________________________________________________

__________________________________________________________________________

1. Did you interact with the tool during Pilot 3, and if so – how?

__________________________________________________________________________

__________________________________________________________________________

1. Do you believe you had enough time to interact with the tool to be able to provide the feedback requested?

• Yes • No

1. How would you describe the decision-support offered by INMOS, compared to what you would normally have used?

__________________________________________________________________________

__________________________________________________________________________

1. **Value of functionalities**

What is your ***believed* value** of the different functions below?
Mark your rating on each row in the scale. (1=no value at all – 7=very extensive value

| No value at all | 1 | 2 | 3 | 4 | 5 | 6 | 7 | Very extensive value |
| --- | --- | --- | --- | --- | --- | --- | --- | --- |
| 1. See resources (e.g. personnel and UxVs) on the map | O | O | O | O | O | O | O |  |
| 1. See victims on the map | O | O | O | O | O | O | O |  |
| 1. See objects (e.g. rubble, smoke, signs) on the map | O | O | O | O | O | O | O |  |
| 1. See areas with toxic gases on the map | O | O | O | O | O | O | O |  |
| 1. Getting route suggestions according to agent (e.g. personnel, UxV) capacity | O | O | O | O | O | O | O |  |
| 1. Getting recommended missions with several possibilities for agents, paths, etc. | O | O | O | O | O | O | O |  |
| 1. Dynamic map updates based information from UxVs | O | O | O | O | O | O | O |  |
| 1. Getting sensor data (e.g. IR and RGB streams) from UxVs | O | O | O | O | O | O | O |  |
| 1. Getting 3D data based information from UxVs | O | O | O | O | O | O | O |  |
| 1. Manually operated UxVs | O | O | O | O | O | O | O |  |
| 1. Autonomous UxVs | O | O | O | O | O | O | O |  |
| 1. UxV working together to execute a mission | O | O | O | O | O | O | O |  |
| 1. A tactical communication system | O | O | O | O | O | O | O |  |
| k) own suggestion | O | O | O | O | O | O | O |  |
| l) own suggestion | O | O | O | O | O | O | O |  |

**Comments on INTREPID Tools**

1. **Is there any of the INTREPID tools you think would need extensive modification for being useful in operations?** _______________________________________________________________

____________________________________________________________________________

____________________________________________________________________________

1. **Is there anything you are missing among the INTREPID tools?** ____________________________________________________________________________

____________________________________________________________________________

____________________________________________________________________________

____________________________________________________________________________

**Your view on the Pilot 3 scenario**

1. What is your opinion on the Pilot 3 scenario? Mark your rating 1-7 on each row of the scale.

| Not representative / unrealistic | 1 | 2 | 3 | 4 | 5 | 6 | 7 | Representative / realistic |
| --- | --- | --- | --- | --- | --- | --- | --- | --- |
| The scenario is something we could be set to handle | O | O | O | O | O | O | O |  |
| The scenario was useful for demonstrating the INTREPID tools | O | O | O | O | O | O | O |  |
| The scenario was realistic | O | O | O | O | O | O | O |  |
| The scenario was complex enough | O | O | O | O | O | O | O |  |
| The scenario held to much details | O | O | O | O | O | O | O |  |
| *Own suggestion:* | O | O | O | O | O | O | O |  |
| *Own suggestion:* | O | O | O | O | O | O | O |  |

7. **Has there been any change in the patient's health outcomes?**

___________________________________________________________________________

____________________________________________________________________________

____________________________________________________________________________

8. **Has there been any impact of care integration on time?**

___________________________________________________________________________

____________________________________________________________________________

____________________________________________________________________________

9. **Has there been any impact of integration on team coordination?**

___________________________________________________________________________

____________________________________________________________________________

____________________________________________________________________________
